# The adult environment promotes the transcriptional maturation of human iPSC-derived muscle grafts

**DOI:** 10.1038/s41536-024-00360-4

**Published:** 2024-04-04

**Authors:** Sarah B. Crist, Karim Azzag, James Kiley, Ilsa Coleman, Alessandro Magli, Rita C. R. Perlingeiro

**Affiliations:** 1 Lillehei Heart Institute, University of Minnesota, Minneapolis, MN USA; 2 Stem Cell Institute, University of Minnesota, Minneapolis, MN USA; 3 Human Biology Division, Fred Hutchinson Cancer Center, Seattle, WA USA; 4 Present Address: Sanofi, Genomic Medicine Unit, 225 2nd Ave, Waltham, MA 02451 USA

**Keywords:** Muscle stem cells, Induced pluripotent stem cells, Transcriptomics

## Abstract

Pluripotent stem cell (PSC)-based cell therapy is an attractive option for the treatment of multiple human disorders, including muscular dystrophies. While in vitro differentiating PSCs can generate large numbers of human lineage-specific tissue, multiple studies evidenced that these cell populations mostly display embryonic/fetal features. We previously demonstrated that transplantation of PSC-derived myogenic progenitors provides long-term engraftment and functional improvement in several dystrophic mouse models, but it remained unknown whether donor-derived myofibers mature to match adult tissue. Here, we transplanted iPAX7 myogenic progenitors into muscles of non-dystrophic and dystrophic mice and compared the transcriptional landscape of human grafts with respective in vitro-differentiated iPAX7 myotubes as well as human skeletal muscle biospecimens. Pairing bulk RNA sequencing with computational deconvolution of human reads, we were able to pinpoint key myogenic changes that occur during the in vitro–to–in vivo transition, confirm developmental maturity, and consequently evaluate their applicability for cell-based therapies.

## Introduction

Pluripotent stem cells (PSCs) are currently used as a resource for numerous tissue systems in the development of disease-modifying therapies, pre-clinical models and tissue-engineering applications^1^. Current technology allows for efficient differentiation of human PSCs into cell types poised to reconstitute heart^2–6^, skeletal muscle^7–11^, hematopoietic lineages^12–16^, lung^17,18^, liver^19–21^, and more^22–25^. Advancements in PSC-derived skeletal muscle differentiation^7–9,26,27^, purification^8–10,28^ and genome editing^29–33^ in the past two decades have brought the concept of allogeneic (healthy HLA-matched donor) or autologous (patient-derived gene-corrected) stem cell therapies closer to the clinic. To date, engraftment in murine skeletal muscle has been reported upon the transplantation of human myogenic progenitor cells using several different methodologies, including transgene-free^8,9,27^, which make use of defined small molecules, and transgene-dependent^7,10,30^, which utilize overexpression of key transcription factors from the skeletal muscle hierarchy (e.g., PAX7, MYOD). We have previously demonstrated that doxycycline (dox) inducible expression of PAX7 (iPAX7) in differentiating PSCs enables the generation of large quantities of human myogenic progenitors that are able to not only replace damaged myofibers but also sustain long-term engraftment through the contribution to the functional satellite cell pool^7,32–34^.

Unfortunately, despite their potential, many induced PSC (iPSC)-derived lineages still exhibit immature phenotypes that resemble prenatal stages of development when differentiated in vitro^1,6,26^. This bottleneck has the potential to severely impair the clinical application of PSC-based cell therapies since proper tissue function requires the acquisition of adult cellular and molecular structures^1^. A major pursuit in the field, therefore, is to demonstrate that the strategies undertaken in vitro or adaptations occurring in vivo result in the development of sustained mature tissue. Paradoxically, little focus has been placed on characterizing iPSC-derived myofibers. Beyond assessing the size of donor-derived engraftment^7–9^, not much is currently known about just how comparable human iPSC-derived myofibers are to adult human skeletal muscle. To this point, though we previously demonstrated that human iPAX7 myogenic progenitors provide functional improvement in mouse models of Duchenne muscular dystrophy (DMD)^7,10,33^ and limb-girdle muscular dystrophy type 2I (LGMD2I)^34^, it remains unknown whether these donor-derived myofibers have matured into adult tissue.

Skeletal muscle maturation is a complex process that includes many changes in morphology, function, and epigenomic and transcriptome profiles^35,36^. As such, when characterizing the maturation of iPSC-derived myofibers in vivo, metrics such as myofiber number and size, while important, are not sufficient to demarcate a mature fiber from an immature fiber. In mammalian development, myogenesis is highly dependent on extrinsic factors from the environment (e.g., morphogen gradients, signaling molecules, tissue stiffness)^35^. For skeletal muscle, PSC-derived myofiber maturation would be marked by a progression past early lineage regulators (MYF5/MYOD/MYF6/MYOG) as well as a switch from neonatal-specific genes towards postnatal isoforms^36^. As the most fundamental component of muscle, myosin determines the rate of contraction and the resulting metabolic demands of each muscle fiber^37^. Thus, individual myosin heavy chain (MyHC) isoforms are the predominate markers of immature, slow (type I), and fast (type II) skeletal muscle fiber types^37^. During myogenesis, embryonic and slow type I MyHC are expressed first; then, at later stages of maturation, myofibers develop fast-twitch MyHC types IIa, IIb, and IIx^35–37^.

Here we sought to understand the extent of molecular maturation after donor-derived nuclei form new myofibers or are incorporated into existing adult murine myofibers. Does introduction into the adult muscle environment facilitate iPSC-derived myofiber maturation or do iPAX7 myofibers remain embryonic/fetal in character, as their myotubes do in vitro?

To address this question, we transplanted human iPAX7 myogenic progenitors into muscles of unaffected and dystrophic mice and compared the myogenic stage of the resulting donor myofibers with in vitro-differentiated myotubes and human skeletal muscle biospecimens across development. We concluded that advanced myogenic maturation occurs as donor-derived myonuclei form myofibers, thus recapitulating the myogenic phases observed during human development.

## Results

**Fig. 1:**
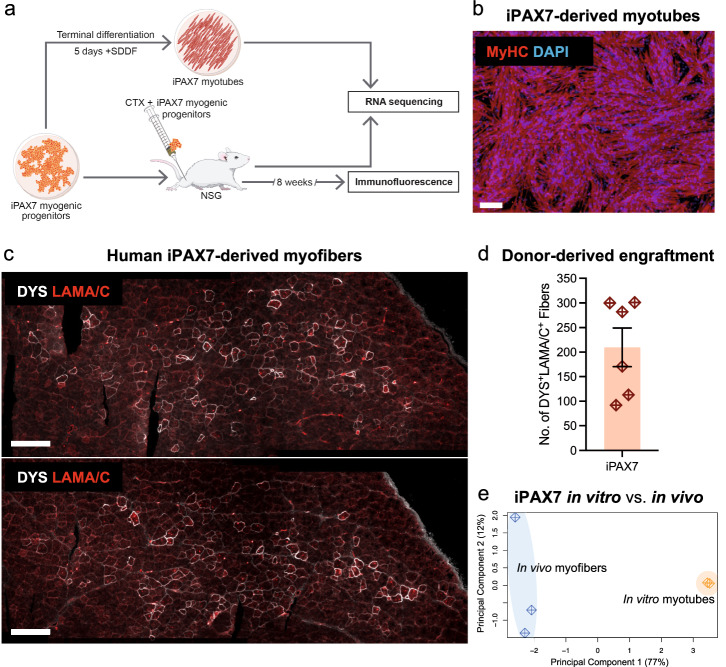
Human iPSC–derived iPAX7 myogenic progenitors give rise to myofibers in vivo that are transcriptionally different from in vitro-differentiated iPAX7 myotubes. **a** Schematic outline of studies. iPAX7 myogenic progenitors were subjected to in vitro terminal differentiation (upper panel) or transplanted into CTX pre-injured muscles of NSG mice (lower panel). Analysis consisted of immunofluorescence staining and RNA-sequencing. **b** Representative image of iPAX7-derived myotubes following in vitro terminal differentiation. MyHC in red, DAPI in blue. Scale bar = 100 µm. **c** Representative images of iPAX7-derived human myofibers (DYS + LAMA/C + ) in transplanted NSG skeletal muscle. LAMA/C = human lamin A/C (red), DYS = human dystrophin (white). Scale bar = 500 µm. **d** Dot-plot measuring the total number of DYS^+^LAMA/C^+^ (donor-derived) myofibers engrafted per NSG muscle (*n* = 6). Error bar represents standard error of the mean (s.e.m.). **e** Principal component analysis (PCA) plot of in vitro-differentiated myotube (*n* = 2) and in vivo myofiber (*n* = 3) samples.

To perform transcriptomic evaluation of myogenic maturation in vitro vs. in vivo, we divided cultures of human iPSC-derived iPAX7 myogenic progenitors into a fraction for in vitro terminal differentiation and a fraction for transplantation into cardiotoxin (CTX)-injured skeletal muscles of NSG mice (Fig. 1a). Following validation that this preparation of iPAX7 myogenic progenitors produced abundant myotube-derivatives in vitro (Fig. 1b) and yielded quality engraftment (Fig. 1c, d), we then proceeded with the RNA sequencing of in vitro and in vivo samples.

### The transcriptional landscape of post-transplant donor-derived human myofibers is very different from in vitro-differentiated myotubes

We performed transcriptome analysis on in vitro*-*differentiated myotubes as well as whole skeletal muscles collected from transplanted NSG mice (Fig. 1a). To restrict our analysis to the signal from donor (e.g., human) cells, human transcripts were extracted using the R-package XenofilteR, which separates human from mouse sequence reads based on the edit-distance between a sequence read and reference genome^38^. Principal component analysis (PCA) of all differentially expressed genes (DEGs) demonstrated that human iPAX7-derived in vitro-differentiated myotubes (referred to as “in vitro myotubes”) and human iPAX7-derived myofibers in NSG mice (referred to as “in vivo myofibers”) display distinct transcriptional profiles (Fig. 1e).

### Maturation signature is enhanced in human in vivo myofibers compared to in vitro myotubes

**Fig. 2:**
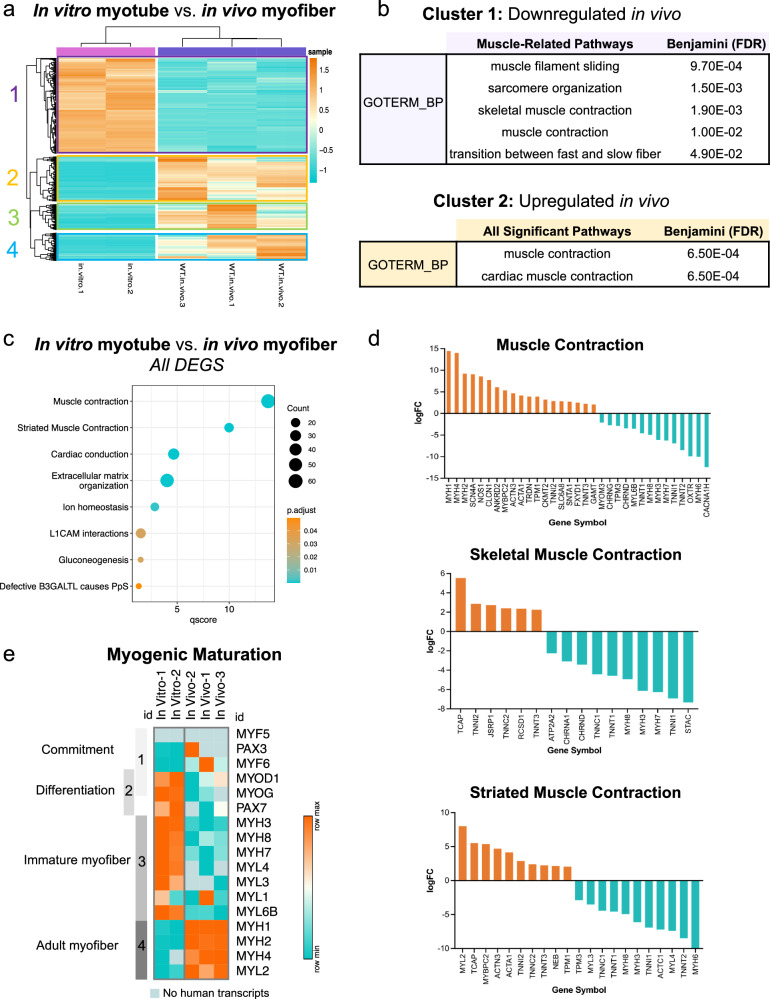
Human donor-derived in vivo myofibers are more transcriptionally mature than in vitro-differentiated myotubes*.* **a** Heatmap displaying expression values (logCPM) for all differentially-expressed genes (DEGs) between in vitro*-*differentiated myotubes and human donor-derived myofibers in NSG mice (“in vivo myofibers”). Hierarchical clustering was performed to group samples and genes by similarity, resulting in four unique clusters of genes arose. High expression in orange, low expression in teal. **b** Tables describing all muscle-related pathways enriched in Cluster 1 and all significant pathways enriched in Cluster 2 after pathway analysis (DAVID, GOTerm Biological Processes). **c** Bubble-plot depicting the top pathways that emerge when all DEGs are included in pathway analysis (Reactome) for in vitro myotubes versus in vivo myofibers. **d** Bar graphs showing the log-fold change (logFC) of genes that are annotated to three different gene sets that emerged as top pathways. Enriched genes in orange and downregulated genes in teal. **e** Heatmap of expression (logCPM) for major myogenic genes in in vitro myotube and in vivo myofiber samples. Gray bars on the left side highlight the development stage associated with each gene (1 - myogenic commitment, 2 – differentiation, 3 – immature myofiber, 4 – adult myofiber). High expression is in orange and low expression in teal, where color is based on row minimums/maximums. Light blue shading represents instances where no human transcripts were measured for that sample (*n* = 2 and *n* = 3 for in vitro myotube and in vivo myofiber samples, respectively).

To understand the differences between in vitro and in vivo samples, all DEGs were plotted in a heatmap with hierarchical clustering (Fig. 2a). Stark patterns of gene expression changes were observed, where cluster 1 genes are significantly downregulated upon engraftment but genes in clusters 2, 3 and 4 are all dramatically enhanced with the in vitro–to–in vivo transition (Fig. 2a). Pathway analysis (DAVID^39^) of the genes with membership in each subset reveals the ongoing changes in myogenesis and muscle-related processes. Cluster 1 highlights the modulation of gene sets involved in muscle filament sliding, skeletal muscle contraction, and the transition between fast and slow fiber (Fig. 2b). Conversely, clusters where gene expression increases with the transition in vivo include terms such as muscle contraction (cluster 2), Z disc (cluster 2), sarcolemma (cluster 2) and extracellular matrix (cluster 4) (Fig. 2b, Supplementary Fig. 1A). Further, when pathway analysis was performed on all DEGs rather than specific subsets, the top pathways altered between the in vitro myotubes and in vivo myofibers are related to muscle structure and function (Fig. 2c), highlighting that the overarching evolution from in vitro to in vivo is myogenic in nature.

To look more closely at the genes driving these signatures, the log-fold change of all genes annotated to each top gene set were measured (Fig. 2d). Here we find both significant gene enrichment and depletion of muscle-specific genes. For example, nebulin (NEB)^40^ and titin-cap (TCAP)^41^, known for providing sarcomeric stability, as well as adult fast-twitch troponin isoforms TNNI2 and TNNC2^42,43^, are significantly upregulated in vivo (Fig. 2d), whereas actin C1 (ACTC1), only expressed during development^44^, is robustly expressed in in vitro myotubes and downregulated in in vivo myofibers (Fig. 2d).

### Adult myosin isoforms become prominent and preferential for donor-derived populations in NSG mice

To specifically compare the maturation status of human in vivo myofibers and in vitro myotubes, we next referenced key myogenic transcription factors involved in developmental myogenesis as well as MyHCs and myosin light chain (MYL) isoforms known to specifically characterize embryonic and adult muscles^45–47^. Comparing in vivo myofibers against in vitro myotubes, we observed a significant decrease in the expression of developmental transcription factors (MYOD1 and MYOG) and immature muscle MyHC and MYL isoforms (MYH3, MYH8, MYL4, MYL6B) concurrent with the increase in mature isoforms (MYH1, MYH2, MYH4) (Fig. 2e). Isoforms encoding for adult slow type I skeletal muscles and fetal muscle^45,46^ demonstrated similar changes, with downregulation of MYH7 and MYL3, respectively and upregulation of MYL2 (Fig. 2e). Taken together, the data demonstrate that developmental maturation of human iPAX7-derived myogenic progenitors occurs as they form myofibers in the adult skeletal muscle environment.

### Human in vitro myotubes closely resemble fetal human muscle whereas donor-derived in vivo myofibers cluster with adult human skeletal muscle

**Fig. 3:**
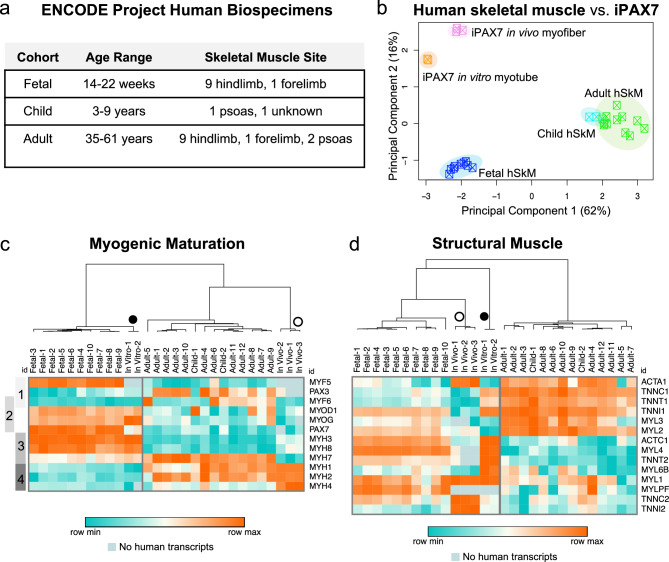
The myogenic signature of in vivo myofibers strongly corresponds with adult human skeletal muscle, whereas in vitro myotubes more closely resemble fetal human muscle. **a** Table depicting the sample information pertaining to the human biospecimens selected from the ENCODE Project database for comparison with iPAX7-derived samples. **b** PCA plot of human skeletal muscle (termed “fetal SkM”, “child SkM”, and “adult SkM”) as well as human in vitro myotubes and in vivo myofibers (*n* = 10 fetal SkM, *n* = 2 child SkM, *n* = 12 adult SkM, *n* = 2 in vitro myotube, and *n* = 3 in vivo myofiber samples). **c** Heatmap of expression (logCPM) for major myogenic genes comparing human skeletal muscle biospecimens (“fetal”, “child” or ”adult”) against in vitro myotube and in vivo myofiber samples. Gray bars on the left side highlight the development stage associated with each gene (1 - myogenic commitment, 2 – differentiation, 3 – immature myofiber, 4 – adult myofiber). High expression is in orange and low expression in teal, where color is based on row minimums/maximums. **d** Heatmap of expression (logCPM) for other muscle-related genes involved in skeletal muscle structure, comparing human skeletal muscle against in vitro myotube and in vivo myofiber samples. High expression is in orange and low expression in teal, where color is based on row minimums/maximums.

To determine how closely human in vivo myofibers resemble their true human counterparts, we compared human in vivo myofiber samples, as well as in vitro myotubes, against human skeletal muscle biospecimens collected throughout development. For this, we used RNA sequencing data of human samples aggregated by the ENCODE Project^48^, and specific samples were chosen based on anatomical site of collection (e.g., skeletal muscle, hindlimb preference) and age (Fig. 3a). We performed standard analysis on fourteen human samples spanning different developmental stages of myogenesis: ten fetal, two child and twelve adult donors (termed “fetal SkM”, “child SkM”, and “adult SkM”, respectively). As shown in Fig. 3b, PCA evidenced that donor-derived samples cluster more closely to each other than to the human ENCODE samples. Child and adult SkM clusters overlapped, suggesting a high degree of transcriptional similarity, whereas fetal SkM clustered the furthest from all samples (Fig. 3b). Because our in vitro and in vivo samples were generated using the same technology, the PCA approach conflates platform-related variance with biological variance. We therefore directly investigated specific gene sets of relevance to the question of developmental maturity and identity of muscle. We found that genes associated with myogenic commitment and differentiation (PAX7, MYF5, MYOD1, MYOG) are highly expressed in the human fetal SkM but not child or adult SkM (Fig. 3c). Expression of embryonic MYH3 and fetal MYH8 genes was also confined to the fetal tissue. Conversely, adult myosin isoforms (MYH1, MYH2) were only expressed in the postnatal human specimens (Fig. 3c). Myogenic genes typically annotated to early myogenic commitment such as PAX3 and MYF6 also showed selective expression in at least half of the postnatal human samples (Fig. 3c).

Comparing these expression patterns side-by-side with human in vitro myotubes and in vivo myofibers, it became clear that in vitro myotubes strongly cluster with fetal SkM whereas the in vivo myofiber counterparts cluster with the adult human muscle. Apart from MYF5 and PAX7 expression, which mark active regeneration, in vitro myotube samples had an identical pattern of myogenic regulation as fetal SkM (Fig. 3c).

Focusing on the in vivo myofibers, we noticed a strong signature of mature muscle. Akin to the adult and child SkM, in vivo myofibers show robust expression of adult myosin isoforms (Fig. 3c). A difference, though, that set human in vivo myofibers apart from adult skeletal muscle included low expression of PAX3 and MYF6 (Fig. 3c). PAX3, typically expressed by paraxial mesoderm progenitors, and MYF6, a regulator of progenitor differentiation, are not abundant in established myofibers of the lower limb. Accordingly, this explains their absence from donor-derived myofibers but not the adult human samples, which represent a mix of fibers and progenitors from a greater breadth of SkM sites.

Interestingly, we also noticed trends in the composition of fiber types in all myofiber populations. MYH4, a postnatal gene encoding type IIB myofibers^45,46^, was only significantly expressed in the in vivo myofibers and not in adult human samples (Fig. 3c). MYL1, encoding for two proteins that form either embryonic muscle or fast-twitch myofibers^45,46^, was seen to be generally expressed across all samples (Fig. 3d). Interestingly, MYL2 and MYL3, genes similarly able to contribute to either fetal muscle and/or fast-twitch type II myofibers^45,46^, had a more selective expression across samples. MYL2 was not expressed in the in vitro myotube samples, yet expression ranged from moderate in the fetal SkM and in vivo myofibers to high levels in the adult and child SkM samples (Fig. 3d). MYL3, on the other hand, was not observed in either the in vivo myofiber or in vitro myotube samples (Fig. 3d). Conversely, MYH7, a gene encoding for slow type I fibers, showed low expression in the in vivo myofibers but was expressed strongly in many adult SkM and to some extent in all other samples (Fig. 3c). Other slow type I myofiber-associated genes such as TNNC1^49^, TNNT1^49^, and TNNI1^49^ were most collectively and abundantly expressed by adult and child SkM, but several were also seen in fetal SkM (TNNI1), and in vitro myotubes (TNNI1/TNNC1/TNNT1) (Fig. 3d). However, these genes were not expressed in the in vivo myofiber samples. Taken together, these data may support that donor-derived myofibers may become type II muscle, the dominant fiber type of the host tibialis anterior, rather than slow type I muscle.

Collectively, these results indicate that the iPAX7-derived myotubes and myofibers achieve remarkable similarity to human myogenesis, despite not all genes being identical. That said, one also needs to remember that while the human in vivo myofibers were formed in response to a tissue injury, it is unknown if the ENCODE biospecimens had any similar event prior to collection. Further, the murine environment has likely also influenced iPAX7-derived populations in ways not found in human muscle regeneration. Thus, it is hard to say how much weight is appropriate to place on these myofiber type differences. Regardless, in sum, we show that advanced myogenic maturation is indeed occurring as donor-derived myonuclei form myofibers in the NSG wild-type (WT) mouse model. Importantly, this pattern largely recapitulates the myogenic stages that occur in human development.

### Post-transplant donor-derived in vivo myofibers also mature in the NSG-*mdx*^4Cv^*mouse* model of DMD

**Fig. 4:**
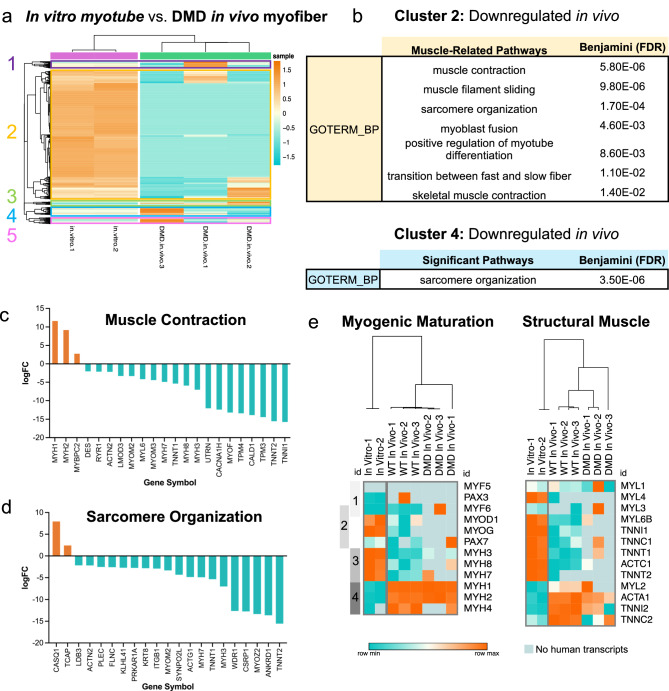
Human donor-derived myofibers engrafted in dystrophic mice are also more transcriptionally mature compared to in vitro-differentiated myotubes. **a** Heatmap displaying expression values (logCPM) for all DEGs between human in vitro myotubes and in vivo myofibers in NSG-mdx^4Cv^ mice (“DMD in vivo myofibers”). Hierarchical clustering was performed to group samples and genes by similarity, which identified five unique clusters of genes arose. High expression in orange, low expression in teal. **b** Tables describing all muscle-related pathways enriched in Cluster 2 and all significant pathways enriched in Cluster 4 after pathway analysis (DAVID, GOTERM Biological Processes). **c**, **d** Bar graphs showing the log-fold change of genes that are annotated to two different gene sets that emerged as top pathways. Enriched genes in orange and downregulated genes in teal. **e** Heatmap of expression (logCPM) for major myogenic genes for in vitro myotubes with DMD in vivo myofibers (left). Gray bars on the left side highlight the development stage associated with each gene (1 - myogenic commitment, 2 – differentiation, 3 – immature myofiber, 4 – adult myofiber). Heatmap of expression (logCPM) for other muscle-related genes involved in skeletal muscle structure, comparing in vitro myotubes with DMD in vivo myofibers (right). High expression is in orange and low expression in teal, where color is based on row minimums/maximums. Light blue shading represents instances where no human transcripts were measured for that sample (*n* = 2 and *n* = 3 for in vitro myotube and DMD in vivo myofiber samples, respectively).

Having established that human iPAX7 myogenic progenitors mature upon transplantation in non-dystrophic muscle, and that this mirrors human myogenesis, we next wanted to assess if the same level of maturation occurred in a muscle environment recapitulating some aspects of human pathology. To address this question, we used dystrophin-deficient immunodeficient (NSG-*mdx*^4Cv^)^50^ mice, a model of DMD, as an additional cohort (Supplementary Fig. 2A). Transcriptomic comparison between in vitro myotubes and in vivo myofibers from the DMD background shows very stark differences, similar to the results observed in the WT muscle (Fig. 4a, Supplementary Fig. 2B). Cluster 2, the major subset in this DEG heatmap, showed reduced gene expression in vivo and included pathways related to muscle contraction, muscle filament sliding, and the transition between fast and slow fibers, similarly to the WT in vivo myofiber dataset (Fig. 4b). However, pathway analysis also implicated other muscle-related terms as affected by these gene changes: sarcomere organization, myoblast fusion, and positive regulation of myotube differentiation (Fig. 4b, Supplementary Fig. 2C). Therefore, we observe large global changes in the myogenic program even in the context of the dystrophic mouse model.

Taking a more granular approach, we again looked at MyHC patterns to determine the developmental maturity of DMD in vivo myofibers compared to in vitro myotubes and WT in vivo myofibers. Similar to the results obtained in the WT background, DMD in vivo myofibers showed reduced expression of immature isoforms, such as MYH3, MYH8, MYL4, MYL6B, and high levels of the postnatal isoforms MYH1 and MYH2 (Fig. 4e). Developmental actin ACTC1 was also not expressed in DMD in vivo myofibers (Fig. 4e). These data suggest that myogenic maturation does occur in iPAX7-derived myofibers formed in dystrophic skeletal muscle despite its altered environment. However, unlike WT in vivo myofibers, the transition between fast and slow fiber isoforms was less pronounced in the DMD setting. MYH7 (slow isoform) and MYH4 (fast isoform) remained unchanged from in vitro to in vivo (Fig. 4e). These more subtle differences between WT and DMD myofiber implies that the identity and thereby function of donor-derived myofibers may differ in the WT and DMD settings.

### Most transcriptional differences between donor-derived myofibers in WT and DMD are not myogenesis-related

**Fig. 5:**
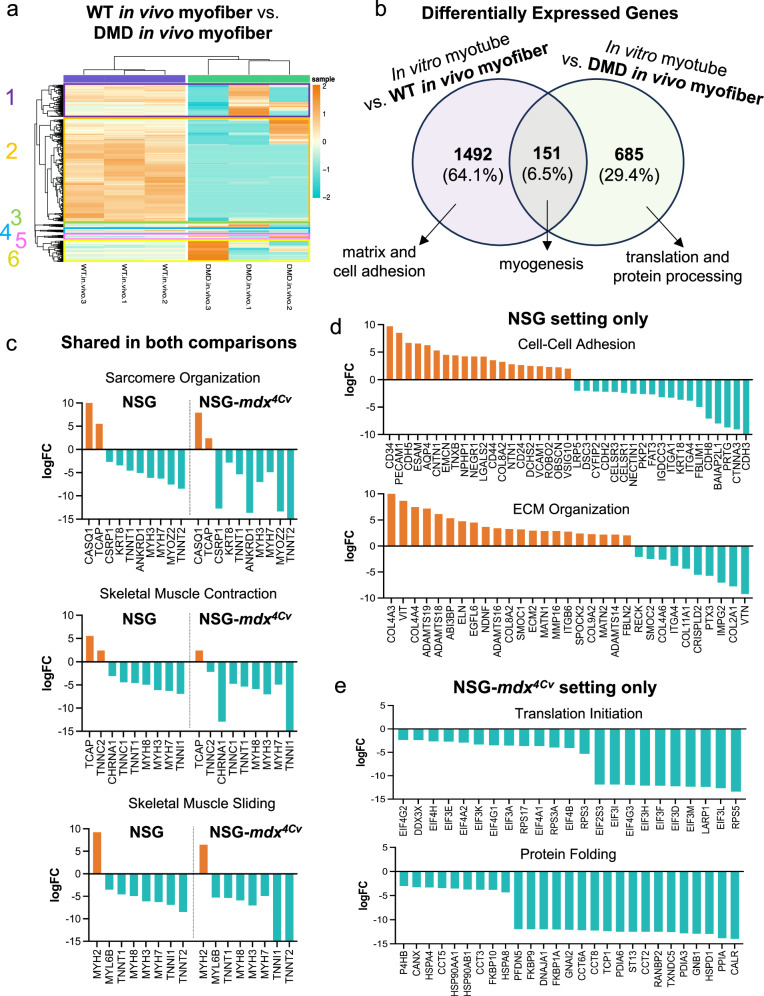
Transcriptional differences between WT and DMD donor-derived myofibers are largely not myogenesis-related. **a** Heatmap displaying expression values (logCPM) for all DEGs WT in vivo myofibers versus DMD in vivo myofibers. Hierarchical clustering was performed to group samples and genes by similarity. In so doing, six unique clusters of genes arose. Genes in all cluster appear to be downregulated in DMD in vivo myofiber samples. High expression in orange, low expression in teal. **b** Venn diagram visualizing the overlap of DEGs between the two comparisons previously made: (i) In vitro myotubes vs. WT in vivo myofibers and (ii) in vitro myotubes vs. DMD in vivo myofibers. **c** Bar graphs showing the logFC of genes that are annotated to top gene sets that shared by the two in-vitro-to-in-vivo comparisons. **d** Bar graphs showing the logFC of genes that are annotated to top gene sets are unique to the in vitro myotube vs. WT in vivo myofiber comparison. **e** Bar graphs showing the logFC of genes that are annotated to top gene sets are unique to the in vitro myotube vs. DMD in vivo myofiber comparison. All bar graphs show only the DEGs that were part of the shared or unique subset, not the annotated geneset in its entirety.

Hierarchical clustering of donor-derived myofibers formed in WT and DMD mice highlighted the extent of differential gene regulation across the two animal models (Fig. 5a, Supplementary Fig. 3A–C). As these data establish significant distinctions, we sought to better understand what might underlie this observation. We first looked at precisely which genes were shared, and which were unique, between our two primary comparisons: (i) in vitro myotubes vs. WT in vivo myofibers and (ii) in vitro myotubes vs. DMD in vivo myofibers (Fig. 5b). Interestingly, we found that most genes related to myogenesis were actually shared between both transplanted in vivo models compared to in vitro myotubes (Fig. 5b, Supplementary Fig. 3D). In fact, top pathways annotated to the shared gene sets included sarcomere organization, skeletal muscle contraction, and skeletal muscle sliding (Fig. 5c). Of note, we observed the same trend in gene expression (log-fold change) in these DEGs, further indicating that the in vivo myofibers are functioning similarly in both settings (WT and DMD). The non-overlapping genes, representing the actual transcriptional differences between the donor-derived populations engrafted in the two mouse models, are annotated to cell-cell adhesion and matrix organization (WT only; Fig. 5d, Supplementary Fig. 3D) or to translation and protein processing (DMD only*;* Fig. 5e, Supplementary Fig. 3D) pathways. This led us to theorize that perhaps interactions with the dystrophic microenvironment might be at play.

In conclusion, the data taken together demonstrate that human iPSC-derived iPAX7 myogenic progenitors developmentally mature into adult-like myofibers in both unaffected and dystrophic skeletal muscle. Since the present study made use of one iPSC line, future studies should confirm whether similar level of maturation is obtained across WT iPSC lines as well as gene-edited DMD iPSCs. Although outside the scope of this study, a thorough interrogation of the interplay between host and donor will be necessary to tease apart the mechanisms that underlie the non-myogenic differences observed in the in vivo myofibers across NSG and NSG-*mdx*^4Cv^ settings.

## Discussion

Here, we sought to determine the maturity status of the skeletal muscle that is generated when iPSC-derived iPAX7 myogenic progenitors are transplanted into WT (NSG) and DMD (NSG-*mdx*^4Cv^) mouse models. Previously we showed that mice receiving iPAX7 cell transplants demonstrated improved muscle contractile function compared to PBS-injected controls, and, upon necropsy, revealed measurable numbers of donor-derived myofibers^7,34^, suggesting functionality of engrafted human muscle. However, it remained unknown to what degree the functional muscle graft retained embryonic or fetal characteristics or took on adult muscle features. To date, this question has not been addressed using any protocol for iPSC muscle derivatives. Taking a bulk RNA sequencing approach paired with computational deconvolution of human reads, we were able to pinpoint key myogenic changes that occur in vivo and demonstrate that iPAX7-derived myofibers are far more adult-like in nature than iPAX7-derived myotubes that are terminally differentiated in vitro. Importantly, the in vivo maturation was largely unaffected in the presence of diseased muscle, as evidenced in the experiments using WT and DMD models.

Additionally, we compared human iPAX7-derived myofibers against publicly-available human skeletal muscle datasets to demonstrate that iPAX7-derived myofibers formed in both WT and DMD mouse muscle in large part match the developmental stage of adult skeletal muscle. Conversely, this comparison also showed that the in vitro-differentiated myotubes more closely resemble fetal human muscle, supporting earlier studies that describe these cells as prenatal-like^8,26^.

Lastly, we looked at how donor-derived myofibers differed between unaffected and dystrophic mouse muscle and learned that, although there are a few significant differences, the myogenic programs are largely comparable. Differing between the two mouse models were pathways like cell adhesion, ECM reorganization, and protein folding. Therefore, we hypothesized that the differentially-regulated non-myogenic pathways were the result of the host microenvironment. High-resolution approaches to mapping the dystrophic muscle environment uncover transcriptional heterogeneity due to the presence of degenerating, regenerating and non-regenerating regions^51,52^. Influenced by these local signaling events, donor-derived cells almost assuredly respond to the perceived needs of their surroundings. For example, Chemello and colleagues reported an increase in transcripts controlling protein degradation in dystrophic myonuclei^51^, where our data show higher expression of protein synthesis genes in donor-derived grafts in DMD muscle. Localized signaling and cell-cell communication has now been shown to regulate both regeneration and disease progression^51–53^. While the differential programs annotated to the donor-derived myofibers in the WT or DMD setting aptly reflect this, our results indicate that myogenic maturation occurs regardless of these differences.

In recent years, numerous studies have recognized that the behavior of muscle progenitors after transplantation could be determined primarily by either cell autonomous factors^7–9,30–34^ or the environment^54,55^. In the case of the PSC-derived stem cell pool, we have previously shown that the environment dominated^55^. Here, we also now suspect that the adult environment instructs the maturation of donor-derived muscle fibers in both NSG and NSG-*mdx*^4Cv^ settings equally. While the field has a wealth of knowledge on how the extracellular milieu impacts satellite cell and engineered muscle progenitor stemness^53,56–58^, fewer studies attempt to understand the impact of the in vivo niche on donor-derived myofibers and long-term engraftment. Further, communication between myofiber and microenvironment is bidirectional, as we now appreciate myofibers themselves also influence their local environment^53,56–58^. Our work suggests that studies addressing the molecular crosstalk between donor-derived myofibers and host skeletal muscle may be beneficial when assessing the scope of therapeutic potential for these cell-based approaches.

In conclusion, this study provides a direct comparison between in vitro-generated PSC-derived human myotubes and their in vivo-engrafted myofiber counterparts, revealing a distinct molecular signature induced by the in vivo environment. Whereas in vitro-myotubes are fetal, human PSC-derived skeletal myogenic progenitors produce mature myofibers upon transplantation, fibers that are comparable to primary human muscle. Although evidence of in vivo maturation has been suggested using targeted RNA probes^59^ or immunofluorescence of select developmental myosins^8,60^, unbiased, whole-genome approaches, such as RNA sequencing, to intentionally characterize the in vivo maturation status of human PSC-derived myofibers has not been done before. Although iPAX3-derived myofiber maturation was assessed in both healthy and dystrophic skeletal muscle, it is important to consider the limitations of using only a single iPSC line before making more generalized claims about the ability of iPSC-derived myogenic progenitors as a whole. Since our data suggest that the skeletal muscle environment appears to be the driver in this maturation process, it is possible that advanced in vivo maturation may also occur in PSC-derived myogenic cells generated by other methodologies as well, however this important point needs to be tested on a case-by-case basis. Importantly, this study begins to set the foundation of in vivo developmental maturity and thereby provides reassurance of the potential therapeutic benefit of iPSC-derived iPAX7 myogenic progenitors.

## Methods

### iPSC culture and myogenic differentiation

For these studies, we used PAX7-induced (iPAX7) myogenic progenitors generated from the human iPSC line TC1133 (Lonza), as previously described by ref. ^33^. iPAX7 myogenic progenitors were either expanded for transplantation or switched to terminal differentiation medium (including SB431542, DAPT, Dexamethasone and Forskolin; “SDDF”; Cayman Chemical)^26^. Myotubes treated with SDDF and differentiated for 5 days were used for RNA sequencing in duplicate. In vitro-differentiated samples display high degree of similarity due to standardized differentiation procedures, thus reducing the need for large sample sizes.

### Mice studies

Animal experiments were carried out according to protocols approved by the University of Minnesota Institutional Animal Care and Use Committee. 6–8-week-old male NSG (JAX, stock number 005557) and NSG-mdx^4Cv^ mice were used for these experiments. One day before intramuscular cell transplantation, target muscles (tibialis anterior and quadriceps) were pre-injured with cardiotoxin (CTX, 15 μl of 10 μM stock; Latoxan), as previously described by ref. ^7^. Prior to cell injection, mice were anesthetized with ketamine/xylazine at 80 mg/kg by intraperitoneal injection. Myogenic progenitors were injected at 1.65 × 10^6^ (resuspended in 15 μl of PBS) using a 26 g Hamilton syringe. As control, contralateral legs were injected with the same volume of PBS. Two months after transplantation muscles were collected for assessment of engraftment by immunofluorescence staining and RNA isolation. For euthanasia, mice were injected intraperitoneally with Avertin (250 mg/kg) followed by cervical dislocation.

### Immunofluorescence staining

Dissected skeletal muscles were embedded in Tissue-Tek O.C.T. compound (Sakura), and snap frozen on isopentane pre-cooled with liquid nitrogen. Cryosections of 14 μm were collected on glass slides, and prior to staining, rehydrated with PBS for 5 min at room temperature. Both cultured cells and muscle cryosections were fixed for 30 min at room temperature with 4% PFA, washed with PBS, permeabilized with 0.3% Triton-X100 (Sigma) in PBS for 15 min at room temperature, washed again with PBS, blocked for 30 min blocking with 3% BSA (Sigma), and subsequently incubated with primary antibodies overnight at 4 °C. Primary antibodies included pan-MyHC (MF20; mouse 1:50, DSHB), human LAMIN A/C (rabbit 1:500, ab108595 Abcam), and human DYSTROPHIN (DYS, mouse 1:50, MANDYS106, DSHB). The next day, cryosections were rinsed with PBS and incubated with Alexa Fluor (Thermo Fisher Scientific) secondary antibodies and 4,6-Diamidino-2-phenylindole (DAPI, Santa Cruz) for 1 h at room temperature. After three PBS washes, sections were dried and mounted with Prolong Gold with DAPI (Invitrogen). Slides were analyzed by confocal microscopy (NikonNiE C2 upright confocal microscope). Image processing and quantification were performed with Fiji software. Merge images of human dystrophin and LAMIN A/C were used to quantify donor-derived fibers. A total of 10–12 cryosections, separated by approximately 460 μm, were analyzed for the quantification of donor-derived myofibers.

### RNA isolation and sequencing

Total RNA was isolated from iPAX7 in vitro-differentiated myotubes (*n* = 3) and homogenized tibialis anterior muscles (*n* = 3 NSG; 3 NSG*mdx*^4Cv^) using TRIzol (Invitrogen) and the PureLink RNA mini kit (Invitrogen, #12183025) with on-column DNaseI treatment following the manufacturer’s recommendations. 500 ng of total RNA was used to for generate unique dual-indexed (UDI) TruSeq stranded mRNA libraries. The libraries were then sequenced with a NovaSeq 6000 (S4) using a 150-bp paired-end run by the University of Minnesota Genomic Center. All samples were sequenced at a depth of 20–30 million reads except for the three iPAX7-derived NSG in vivo samples, which were sequenced at a depth of 200 million reads.

### RNA sequencing analysis

#### iPAX7 samples

Reads were trimmed using Trimmomatic (v0.33)^61^ and quality control on raw reads was performed with FastQC (v0.11.9)^62^ (mean quality score 36) was assessed. STAR (v2.7.3a)^63^ was used to align to human (hg38) and mouse (mm10) reference genomes and subtraction of mouse sequences was performed using XenofilteR (v1.6)^38^. Aligned reads were counted for gene associations against the UCSC genes data base with HTSeq (v0.11.0)^64^. Sequencing reads from PBS-injected control muscles were used to refine the human/mouse deconvolution and were not used in downstream applications. DEGs were identified using edgeR (v3.42.4, negative binomial)^65^. Genes were further filtered based on statistical significance and fold change: *p* < 0.05, FDR *q* < 0.05 and |1.5| log-fold change. During this process, count data was also normalized for library size (counts per million, CPM) and log-transformed (logCPM) using edgeR for graphical representation. Enrichment analysis and gene ontology (GO) were performed by using DAVID^39^ and R packages clusterProfiler (v4.8.2)^66^ and ReactomePA (v1.44.0)^67^. Heatmaps with hierarchical clustering were generated using Morpheus (Broad)^68^. Venn diagrams of overlapping gene sets were made using Venny (v2.1.0)^69^. RNA-sequencing data generated in this study has been deposited at GEO and have the following accession numbers: GSE244521, GSM7818598, GSM7818599, GSM7818600, GSM7818601, GSM7818602, GSM7818603, GSM7818604, GSM7818605, GSM7818606, GSM7818607, GSM7818608, GSM7818609, GSM7818610, GSM7818611.

#### ENCODE human skeletal muscle

RNA sequencing data sets were downloaded from the ENCODE portal^48^ (https://www.encodeproject.org/) with the following identifiers: SRR786775, SRR786795, SRR786796, SRR786797, SRR786755, SRR786788, SRR786794, SRR980475, SRR786767, SRR786768, SAMN01737663, SRR14698277, SRR4240813, SRR4240814, SRR4240822, SRR4240870, SRR4422023, SRR4422107, SRR4422532, SRR14636483, SRR14636496, SRR4240819, SRR4422371, SRR4422372. Data alignment and comparison of expression was analyzed using the CHURP pipeline^70^ through the University of Minnesota Supercomputing Institute (MSI). 150-bp paired-end reads (FASTQ) for all samples were trimmed using Trimmomatic (v0.33)^61^ enabled with the optional “-q” option; 3 bp sliding-window trimming from 3’ end requiring minimum Q30. Quality control on raw sequence data for each sample was performed with FastQC (v0.11.7)^62^. Read mapping was performed via HISAT2 (v2.1.0)^71^ using the human genome (hg38) as reference. Gene quantification was done via featureCounts (v2.0)^72^ for raw read counts. Read count data was then normalized for library size (counts per million, CPM) and log-transformed (logCPM) using edgeR (v3.42.4)^65^ for graphical representation. Heatmaps with hierarchical clustering were generated using Morpheus (Broad)^68^.

### Statistical analysis

Data are presented as mean ± SEM. Statistical analysis for the bar-graph in Fig. 1 was not performed since there was only one variable. Statistical analysis for the RNA sequencing datasets was performed through edgeR (v3.42.4)^65^, clusterProfiler (v4.8.2)^66^ and ReactomePA (v1.44.0)^67^ default parameters.

### Reporting summary

Further information on research design is available in the Nature Research Reporting Summary linked to this article.

### Supplementary information

Supplemental Figures

Reporting Summary

## Data Availability

Further information and requests for resources and reagents should be directed to and will be provided by the lead contact, R.C.R.P. (perli032@umn.edu). RNA-sequencing data generated in this study has been deposited at GEO and are publicly available as of the date of publication. Accession numbers are as follows: GSE244521, GSM7818598, GSM7818599, GSM7818600, GSM7818601, GSM7818602, GSM7818603, GSM7818604, GSM7818605, GSM7818606, GSM7818607, GSM7818608, GSM7818609, GSM7818610, GSM7818611. Confocal and inverted microscopy raw data files reported in this paper will be shared by the lead contact upon request. Any additional information required to reanalyze the data reported in this paper is available by the lead contact upon request.

## Code availability

This paper does not report original code. However, any additional information required to reanalyze the data reported in this paper is available by the lead contact upon request.
