# Supplementary material for: The adult environment promotes the transcriptional maturation of human iPSC-derived muscle grafts
Source: NPJ Regen Med. 2024 Apr 4;9:16. doi: 10.1038/s41536-024-00360-4 (PMC10994941; doi:10.1038/s41536-024-00360-4)
Supplement: Supplementary file 1 — Supplemental Figures [file 41536_2024_360_MOESM1_ESM.pdf]

Supplementary Figure 1.

a *In vitro* myotube vs. WT *in vivo* myofiber
Cluster 3

All Pathways (GO:BP)	Benjamini (FDR)
angiogenesis	*6.10E-02**
aerobic respiration	*6.10E-02**

In vitro myotube vs. WT *in vivo* myofiber
Cluster 4

All Pathways (GO:BP)	Benjamini (FDR)
muscle contraction	*1.00E+00**
extracellular matrix organization	*1.00E+00**
cell adhesion	*1.00E+00**
response to hypoxia	*1.00E+00**
positive regulation of angiogenesis	*1.00E+00**

Supplementary Figure 1. Minor DEG subsets are related to non-myogenic processes when comparing human iPSC-derived iPAX7 *in vitro* myotube and WT *in vivo* myofiber samples. A) Tables describing all pathways enriched in Clusters 3 and 4 after pathway analysis (DAVID, GOTERM Biological Processes) for human *in vitro* myotubes *vs.* WT *in vivo* myofibers. No pathways are statistically significant (FDR > 0.05).

Supplementary Figure 2.

a

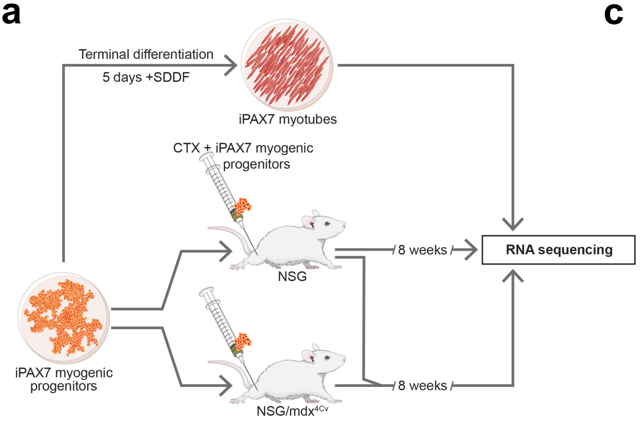

b

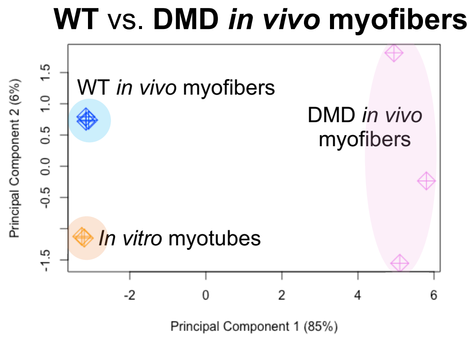

c

In vitro myotube vs. DMD *in vivo* myofiber
Cluster 1

All Pathways (GO:BP)	Benjamini (FDR)
cytoplasmic translation	5.10E-15
translation	6.20E-11

In vitro myotube vs. DMD *in vivo* myofiber
Cluster 3

All Pathways (GO:BP)	Bejamini (FDR)
heart development	*1.00E+00**
endochondral ossification	*1.00E+00**
blood vessel development	*1.00E+00**
skeletal muscle tissue development	*1.00E+00**
cellular response to transforming growth factor beta stimulus	*1.00E+00**

In vitro myotube vs. DMD *in vivo* myofiber
Cluster 5

All Pathways (GO:BP)	Benjamini (FDR)
cytoplasmic translation	8.40E-14
translation	9.20E-12
translational initiation	1.10E-02
skeletal muscle contraction	*7.90E-02**

Supplementary Figure 2. Human myofibers in DMD mice are distinct and several DEG subclusters are non-myogenic when compared to *in vitro* myotubes. A) Schematic outline of studies. iPAX7 myogenic progenitors were subjected to *in vitro* terminal differentiation (upper panel) or transplanted into CTX pre-injured muscles of NSG (WT) or NSG-mdx^{4Cv} (DMD) mice (lower panel). Analysis consisted of RNA-sequencing. B) PCA plot of *in vitro*-differentiated myotubes and *in vivo* myofibers formed in WT and DMD mice (n = 2 *in vitro* myotube, n = 3 WT *in vivo* myofiber, and n = 3 DMD *in vivo* myofiber samples). C) Tables describing all pathways enriched in Clusters 1, 3 and 5 after pathway analysis (DAVID, GOTERM Biological Processes) for *in vitro* myotubes *vs.* DMD *in vivo* myofibers. No pathways in Cluster 3 are statistically significant (FDR > 0.05).

Supplementary Figure 3.

a WT *in vivo* myofiber vs. DMD *in vivo* myofiber
Cluster 1

Top 20 Pathways (GO:BP)	Benjamini (FDR)
cytoplasmic translation	1.80E-29
translation	1.80E-20
mitochondrial ATP synthesis coupled proton transport	1.60E-07
aerobic respiration	2.40E-06
regulation of mRNA stability	5.30E-05
hydrogen ion transmembrane transport	5.60E-05
proteasome-mediated ubiquitin-dependent protein catabolic process	9.40E-05
actin cytoskeleton organization	2.50E-04
collagen fibril organization	2.60E-04
supramolecular fiber organization	4.60E-04
ATP synthesis coupled proton transport	1.50E-03
cellular respiration	3.50E-03
mitochondrial electron transport, ubiquinol to cytochrome c	5.20E-03
muscle contraction+	5.40E-03
mitochondrial electron transport, NADH to ubiquinone	6.70E-03
mitochondrial respiratory chain complex I assembly	8.50E-03
angiogenesis	8.50E-03
muscle cell cellular homeostasis+	8.60E-03
positive regulation of focal adhesion assembly	8.60E-03

b WT *in vivo* myofiber vs. DMD *in vivo* myofiber
Cluster 2

All Significant Pathways (GO:BP)	Benjamini (FDR)
sarcomere organization+	4.10E-08
translation	2.00E-05
muscle contraction+	2.00E-05
mitochondrial ATP synthesis coupled proton transport	4.90E-03
aerobic respiration	5.10E-03
mitochondrial electron transport, NADH to ubiquinone	8.40E-03
cytoplasmic translation	1.80E-02
establishment of protein localization to mitochondrial membrane	2.90E-02
positive regulation of phosphoprotein phosphatase activity	3.70E-02
cellular respiration	3.90E-02
regulation of cytokinesis	3.90E-02
positive regulation of protein serine/threonine kinase activity	3.90E-02
translational elongation	4.60E-02
negative regulation of calcium ion export from cell	4.60E-02
positive regulation of cyclic-nucleotide phosphodiesterase activity	4.60E-02
glycogen biosynthetic process	4.60E-02

c WT *in vivo* myofiber vs. DMD *in vivo* myofiber
Cluster 6

All Significant Pathways (GO:BP)	Benjamini (FDR)
cytoplasmic translation	5.10E-14
translation	8.00E-09
mitochondrial electron transport, cytochrome c to oxygen	8.10E-05
cellular respiration	7.30E-04
mitochondrial ATP synthesis coupled proton transport	5.10E-03
hydrogen ion transmembrane transport	6.70E-03
**sarcomere organization+**	8.60E-03
**muscle contraction+**	1.40E-02
substantia nigra development	1.40E-02
**skeletal muscle contraction+**	3.40E-02

d **Shared** between **"*in vitro* vs. WT myofiber"** and **"*in vitro* vs. DMD myofiber"**

Top Pathways (GO:BP)	Benjamini (FDR)
muscle contraction	2.00E-09
skeletal muscle contraction	2.90E-09
sarcomere organization	2.90E-09
muscle filament sliding	2.90E-09
cardiac muscle contraction	1.40E-07
transition between fast and slow fiber	6.40E-05

"*in vitro* vs. WT in vivo myofiber" only

Top Pathways (GO:BP)	Benjamini (FDR)
cell-cell adhesion	2.20E-05
cell adhesion	2.20E-05
axon guidance	1.70E-04
cardiac muscle contraction	6.00E-04
response to hypoxia	2.00E-03
positive regulation of gene expression	3.10E-03

"*in vitro* vs. DMD in vivo myofiber" only

Top Pathways (GO:BP)	Benjamini (FDR)
cytoplasmic translation	3.60E-90
translation	1.50E-59
translational initiation	2.20E-15
ribosomal small subunit biogenesis	2.30E-11
positive regulation of translation	3.30E-08
collagen fibril organization	4.00E-08

Supplementary Figure 3.

Supplementary Figure 3. Transcriptional program differences between donor-derived myofibers in the WT and DMD settings are largely non-myogenic in nature. Tables describing all pathways enriched in Cluster 1 (A), Cluster 2 (B) and Cluster 6 (C) after pathway analysis (DAVID, GOTerm Biological Processes) for WT *in vivo* myofiber *vs.* DMD *in vivo* myofiber. D) Tables describing all pathways enriched after pathway analysis (DAVID, GOTerm Biological Processes) when analyzing i) genes that are shared between "*in vitro* myotube *vs.* WT *in vivo* myofiber" and "*in vitro* myotube vs. DMD *in vivo* myofiber", ii) genes that are unique to *the "in vitro* myotube *vs.* WT *in vivo* myofiber" comparison and iii) genes that are unique to the "*in vitro* myotube *vs.* DMD *in vivo* myofiber" comparison
