# Supplementary material for: The adult environment promotes the transcriptional maturation of human iPSC-derived muscle grafts
Source: NPJ Regen Med. 2024 Apr 4;9:16. doi: 10.1038/s41536-024-00360-4 (PMC10994941; doi:10.1038/s41536-024-00360-4)
Supplement: Supplementary file 2 — Reporting Summary [file 41536_2024_360_MOESM2_ESM.pdf]

nature portfolio

Corresponding author(s): Rita Perlingeiro, Alessandro Magli

Last updated by author(s): Mar 6, 2024

Reporting Summary

Nature Portfolio wishes to improve the reproducibility of the work that we publish. This form provides structure for consistency and transparency in reporting. For further information on Nature Portfolio policies, see our Editorial Policies and the Editorial Policy Checklist.

Statistics

For all statistical analyses, confirm that the following items are present in the figure legend, table legend, main text, or Methods section.

n/a	Confirmed	
☐	☒	The exact sample size (*n*) for each experimental group/condition, given as a discrete number and unit of measurement
☐	☒	A statement on whether measurements were taken from distinct samples or whether the same sample was measured repeatedly
☐	☒	The statistical test(s) used AND whether they are one- or two-sided<br>*Only common tests should be described solely by name; describe more complex techniques in the Methods section.*
☒	☐	A description of all covariates tested
☒	☐	A description of any assumptions or corrections, such as tests of normality and adjustment for multiple comparisons
☐	☒	A full description of the statistical parameters including central tendency (e.g. means) or other basic estimates (e.g. regression coefficient) AND variation (e.g. standard deviation) or associated estimates of uncertainty (e.g. confidence intervals)
☒	☐	For null hypothesis testing, the test statistic (e.g. *F*, *t*, *r*) with confidence intervals, effect sizes, degrees of freedom and *P* value noted<br>*Give P values as exact values whenever suitable.*
☒	☐	For Bayesian analysis, information on the choice of priors and Markov chain Monte Carlo settings
☒	☐	For hierarchical and complex designs, identification of the appropriate level for tests and full reporting of outcomes
☒	☐	Estimates of effect sizes (e.g. Cohen's *d*, Pearson's *r*), indicating how they were calculated

Our web collection on statistics for biologists contains articles on many of the points above.

Software and code

Policy information about availability of computer code

Data collection	Engraftment: Nikon confocal microscope, NIS element software, Zen upright microscope, Zen 2.3 pro software. RNAseq: NovaSeq 6000 (S4)
Data analysis	Engraftment: Fiji (ImageJ 2.1.0), GraphPad Prism 9. RNAseq: Trimmomatic (v0.33), FastQC (v0.11.9), STAR (v2.7.3a), XenofilteR (v1.6), HTSeq (v0.11.0), edgeR (v3.42.4), DAVID pathway analysis, R packages: clusterProfiler (v4.8.2) and ReactomePA (v1.44.0), Morpheus (Broad), Venny (v2.1.0), University of Minnesota's CHURP pipeline, HISAT2 (v2.1.0), featureCounts (v2.0)

For manuscripts utilizing custom algorithms or software that are central to the research but not yet described in published literature, software must be made available to editors and reviewers. We strongly encourage code deposition in a community repository (e.g. GitHub). See the Nature Portfolio guidelines for submitting code & software for further information.

Data

Policy information about availability of data

All manuscripts must include a data availability statement. This statement should provide the following information, where applicable:

- Accession codes, unique identifiers, or web links for publicly available datasets
- A description of any restrictions on data availability
- For clinical datasets or third party data, please ensure that the statement adheres to our policy

RNA-sequencing data generated in this study has been deposited at GEO and are publicly available as of the date of publication. Accession numbers are as follows: GSE244521, GSM7818598, GSM7818599, GSM7818600, GSM7818601, GSM7818602, GSM7818603, GSM7818604, GSM7818605, GSM7818606, GSM7818607,

GSM7818608, GSM7818609, GSM7818610, GSM7818611. Confocal and inverted microscopy raw data files reported in this paper will be shared by the lead contact upon request. Any additional information required to reanalyze the data reported in this paper is available by the lead contact upon request. This paper does not report original code.

Research involving human participants, their data, or biological material

Policy information about studies with human participants or human data. See also policy information about sex, gender (identity/presentation), and sexual orientation and race, ethnicity and racism.

Reporting on sex and gender	N/A
Reporting on race, ethnicity, or other socially relevant groupings	N/A
Population characteristics	N/A
Recruitment	N/A
Ethics oversight	N/A

Note that full information on the approval of the study protocol must also be provided in the manuscript.

Field-specific reporting

Please select the one below that is the best fit for your research. If you are not sure, read the appropriate sections before making your selection.

☒ Life sciences ☐ Behavioural & social sciences ☐ Ecological, evolutionary & environmental sciences

For a reference copy of the document with all sections, see nature.com/documents/nr-reporting-summary-flat.pdf

Life sciences study design

All studies must disclose on these points even when the disclosure is negative.

Sample size	Sample size was determined to ensure the robustness of the results observed. For engraftment analysis, at least 6 muscles were assessed per mouse cohort, with multiple slides examined per tissue upon immunostaining. Immunostaining was also performed on at least 3 independent myotube (in vitro) replicates. RNAsequencing and downstream analysis was performed on two independent myotube (in vitro) replicates and 3 in vivo muscle samples per mouse cohort.
Data exclusions	No data were excluded from analysis.
Replication	To verify the reproducibility of the experimental findings, we performed all experiments in triplicate.
Randomization	Transplantation studies using NSG and NSGmdx mice were randomized for PBS or cell-treatment per cohort.
Blinding	Blinding was not performed in the animal study due to inherent differences in the preparation of the PBS and cell vials. However, RNA sequencing and downstream analysis was performed in an unbiased manner due to the nature of the workflow and use of existing software platforms. Code was not modified to manipulate the data to favor one group or another.

Reporting for specific materials, systems and methods

We require information from authors about some types of materials, experimental systems and methods used in many studies. Here, indicate whether each material, system or method listed is relevant to your study. If you are not sure if a list item applies to your research, read the appropriate section before selecting a response.

Materials & experimental systems

n/a	Involved in the study	
☐	☒	Antibodies
☐	☒	Eukaryotic cell lines
☒	☐	Palaeontology and archaeology
☐	☒	Animals and other organisms
☒	☐	Clinical data
☒	☐	Dual use research of concern
☒	☐	Plants

Methods

n/a	Involved in the study	
☒	☐	ChIP-seq
☒	☐	Flow cytometry
☒	☐	MRI-based neuroimaging

Antibodies

Antibodies used	Primary antibodies included pan-MyHC (MF20; mouse 1:50, DSHB), human LAMIN A/C (rabbit 1:500, ab108595 Abcam), and human DYSTROPHIN (DYS, mouse 1:50, MANDYS106, DSHB). Alexa Fluor (Thermo Fisher Scientific) secondary antibodies and 4,6-Diamidino-2-phenylindole (DAPI, Santa Cruz) were also used.
Validation	All of these antibodies are commerically available and have been widely used and characterized by the scientific community.

Eukaryotic cell lines

Policy information about cell lines and Sex and Gender in Research

Cell line source(s)	For these studies, we used PAX7-induced (iPAX7) myogenic progenitors generated from the human iPSC line TC1133 (Lonza), as previously described by Kim et al. (Kim H.K. et al. Genomic Safe Harbor Expression of PAX7 for the Generation of Engraftable Myogenic Progenitors. Stem Cell Reports 16(1):10-19 (2021).
Authentication	We have validated this line by whole-genome sequencing and HLA haplotype.
Mycoplasma contamination	This cell line is regularly tested for myocplasma contamination and appears negative.
Commonly misidentified lines (See ICLAC register)	N/A

Animals and other research organisms

Policy information about studies involving animals; ARRIVE guidelines recommended for reporting animal research, and Sex and Gender in Research

Laboratory animals	6–8-week-old male NSG (JAX, stock number 005557) and NSG-mdx4Cv mice (derived in this manuscript: Arpke, R.W. et al. A new immune-, dystrophin-deficient model, the NSG-mdx(4Cv) mouse, provides evidence for functional improvement following allogeneic satellite cell transplantation. Stem Cells 31(8):1611-20 (2013))
Wild animals	N/A
Reporting on sex	N/A
Field-collected samples	N/A
Ethics oversight	Animal experiments were carried out according to protocols approved by the University of Minnesota Institutional Animal Care and Use Committee.

Note that full information on the approval of the study protocol must also be provided in the manuscript.

Plants

Seed stocks	N/A
Novel plant genotypes	N/A
Authentication	N/A
